# Expanded Gene Panel Use for Women With Breast Cancer: Identification and Intervention Beyond Breast Cancer Risk

**DOI:** 10.1245/s10434-017-5963-7

**Published:** 2017-08-01

**Authors:** Erin O’Leary, Daniela Iacoboni, Jennifer Holle, Scott T. Michalski, Edward D. Esplin, Shan Yang, Karen Ouyang

**Affiliations:** 1400 16th Street, San Francisco, CA 94103 USA

## Abstract

**Background:**

Clinicians ordering multi-gene next-generation sequencing panels for hereditary breast cancer risk have a variety of test panel options. Many panels include lesser known breast cancer genes or genes associated with other cancers. The authors hypothesized that using broader gene panels increases the identification of clinically significant findings, some relevant and others incidental to the testing indication. They examined clinician ordering patterns and compared the yield of pathogenic or likely pathogenic (P/LP) variants in non-*BRCA* genes of female breast cancer patients.

**Methods:**

This study analyzed de-identified personal and family histories in 1085 breast cancer cases with P/LP multi-gene panel findings in non-*BRCA* cancer genes and sorted them into three groups by the panel used for testing: group A (breast cancer genes only), group B (commonly assessed cancers: breast, gynecologic, and gastrointestinal), and group C (a more expanded set of tumors). The frequency of P/LP variants in genes with established management guidelines was compared and evaluated for consistency with personal and family histories.

**Results:**

This study identified 1131 P/LP variants and compared variants in clinically actionable genes for breast and non-breast cancers. Overall, 91.5% of these variants were in genes with management guidelines. Nearly 12% were unrelated to personal or family history.

**Conclusion:**

Broader panels were used for 85.6% of our cohort (groups B and C). Although pathogenic variants in non-*BRCA* genes are reportedly rare, the study found that most were in clinically actionable genes. Expanded panel testing improved the identification of hereditary cancer risk. Small, breast-limited panels may miss clinically relevant findings in genes associated with other heritable cancers.

**Electronic supplementary material:**

The online version of this article (doi:10.1245/s10434-017-5963-7) contains supplementary material, which is available to authorized users.

Breast cancer is a multifactorial disease caused by a combination of environmental and genetic factors.^1^ In an estimated 10% of breast cancers, the genetic factor is contributed primarily by mutations in a single gene.^2^ Testing for *BRCA1* and *BRCA2* (*BRCA1/2*) has been available for more than two decades, and multiple professional organizations have developed guidelines for testing individuals based on suggestive personal or family history.^3^–^7^

The clinical impact and cancer risks for individuals with pathogenic variants in *BRCA1/2* are well studied.^8^ Likewise, medical management guidelines have been established and are frequently updated.^3^–^5^ These guidelines also address high- and moderate-risk genes such as *TP53* and *PTEN*.^3^,^9^,^10^ Although medical management guidelines exist for many other genes such as *PALB2*, *CHEK2*, and *ATM*, no defined testing criteria have been established.

The advent of next-generation sequencing (NGS) has allowed for the efficient, cost-effective analysis of many genes simultaneously.^11^–^13^ Multiple studies have demonstrated that compared with multi-gene NGS panels, traditional testing of *BRCA1/2* alone misses potentially actionable findings in a substantial proportion of cases.^14^–^18^ The National Comprehensive Cancer Network (NCCN) and the American Society of Breast Surgeons (ASBS) address the benefits and limitations of multi-gene panels, but they do not explicitly advocate for or against the use this testing method.^3^,^5^ Identified benefits include the increased yield of positive findings, whereas limitations may include higher rates for variants of unknown significance and findings with no established management guidelines.^3^,^5^ In the absence of guidelines for choosing appropriately from the increasing variety of panels, oncology providers must determine whether to select high-risk panels limited to genes with established medical management guidelines or larger panels that have dozens of genes associated with a variety of non-breast hereditary cancers.

Using a consecutive series of 20,592 women with breast cancer undergoing hereditary genetic testing in our commercial laboratory, we sought to clarify the current use of larger hereditary cancer panels and determine their clinical impact. We analyzed a large cohort of breast cancer patients identified as having one or more pathogenic or likely pathogenic (P/LP) variants in genes other than *BRCA1/2*. Clinician ordering patterns were reviewed, and their impact on the rate of P/LP findings was described. Personal and family history information was reviewed to determine how often genetic test results were consistent with the indications for testing and to define the utility of hereditary gene panels for identifying clinically actionable findings. We hypothesized that using broader gene panels increases the identification of clinically significant findings, some relevant and others incidental to the testing indication.

## Methods

The study cohort consisted of all patients referred to our commercial laboratory between February 2015 and August 2016 for diagnostic multi-gene panel testing for hereditary breast and ovarian cancer (HBOC). Patients referred to undergo family variant testing for a known pathogenic mutation were excluded.

The gene panels analyzed for each patient were chosen at the discretion of the ordering provider and ranged from 2 to 79 genes. The price of a panel was the same regardless of the number of genes, making test selection dependent only on patient and provider preferences. Testing was performed with NGS, as previously described,^18^ and variant interpretation was carried out based on an expansion of the American College of Medical Genetics and Genomics (ACMG) guidelines.^19^

**Fig. 1:**
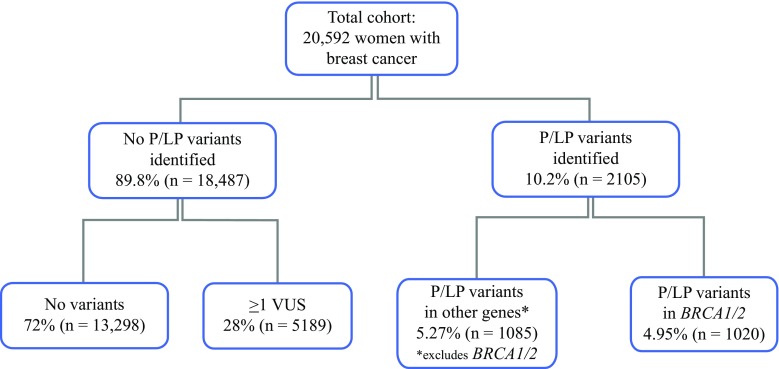
Stratification of patients by variant type. Variants were identified in the patient cohort and categorized according to variant classification and BRCA1/2 status. *n* number of patients, *P/LP* pathogenic/likely pathogenic, *VUS* variant of uncertain significance

A total of 2105 individuals with P/LP variants were identified. Of these individuals, 1020 had P/LP variants in *BRCA1* or *BRCA2* and were excluded, leaving 1085 individuals with P/LP findings in other genes associated with increased risk of heritable cancer (Fig. 1). Notably, common and low-penetrance variants (e.g., *APC* I1370 K, *CHEK2* I157T) were included for analysis.

According to an institutional review board (IRB)-approved study protocol, de-identified personal and family history information from submitted requisition forms and medical records (when available) for each case were reviewed and compared with published testing guidelines^3^ to determine whether the findings were consistent with the reported history information (expected findings) or not (incidental findings). All variants were analyzed to determine the frequency of findings in genes with published medical management guidelines,^4^,^5^ including current ASBS guidelines, NCCN guidelines for HBOC and hereditary colorectal cancer, and other published professional guidelines (see Appendix 1 in Electronic Supplementary Material).

The case series was divided into three primary order groups according to the type of panel ordered:

- Group A: panels consisting of genes associated primarily with breast cancer (*ATM*, *BARD1*, *BRCA1*, *BRCA2*, *BRIP1*, *CDH1*, *CHEK2*, *FANCC*, *MRE11A*, *NBN*, *NF1*, *PALB2*, *PTEN*, *STK11*, *TP53*), most of which have established clinical management guidelines. Orders in this category included up to 15 genes.
- Group B: panels containing all the genes in group A plus genes associated with other commonly assessed cancer types (i.e., breast, gynecologic, and gastrointestinal) (*APC*, *AXIN2*, *BMPR1A*, *CDKN2A*, *DICER1*, *EPCAM*, *GREM1*, *KIT*, *MEN1*, *MLH1*, *MSH2*, *MSH6*, *MUTYH*, *PDGFRA*, *PMS2*, *POLD1*, *POLE*, *RAD51C, RAD51D*, *SDHA*, *SDHB*, *SDHC*, *SDHD*, *SMAD4*, *SMARCA4*, *TSC1*, *TSC2*, *VHL*). Orders in this category included up to 42 genes.
- Group C: large, comprehensive panels including all the genes in groups A and B plus an expanded list of genes for other tumor types (i.e., prostate, sarcoma, brain). Orders in this category included up to 79 genes.

## Results

**Fig. 2:**
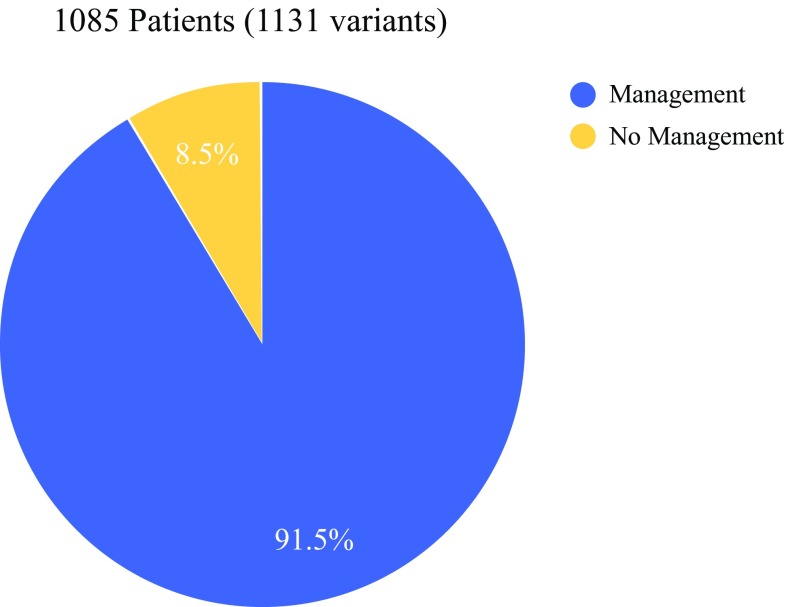
Percentage of identified pathogenic and likely pathogenic variants in the total cohort of genes with medical management guidelines

Among the 1085 cases reviewed, 1131 P/LP variants were identified, with 44 patients (4%) having two or more P/LP variants. Overall, 91.5% of the variants identified in this cohort were in genes with medical management guidelines (Fig. 2).

**Fig. 3:**
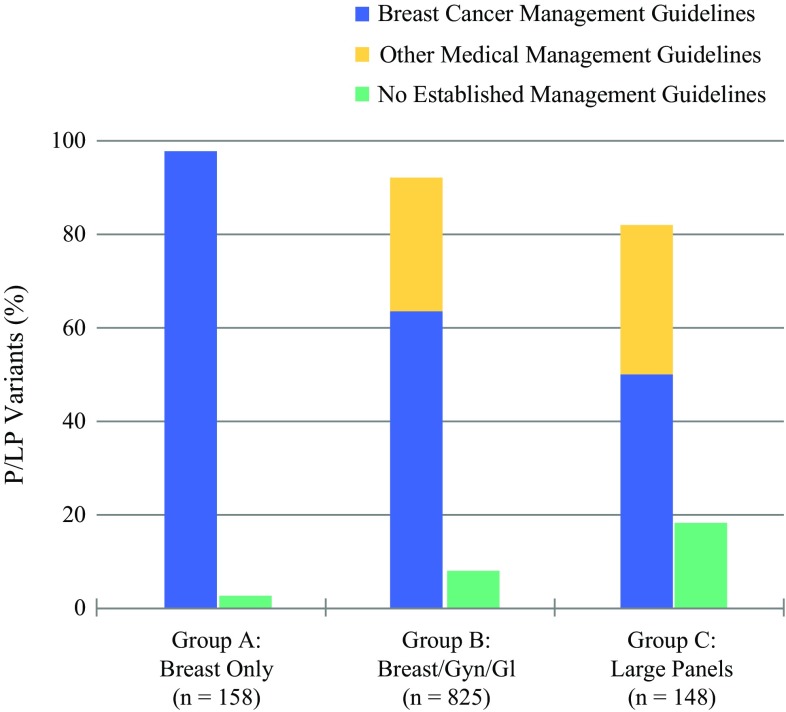
Comparison of P/LP variants and associated management guidelines. *P/LP* pathogenic/likely pathogenic, *GI* gastrointestinal, *Gyn* gynecologic

As shown in Fig. 3, the majority of cases (72.6%) were in ordering group B. As expected, a greater proportion of P/LP variants in genes with breast management guidelines was in group A (97.5%) than in groups B (63.6%) and C (50%). In groups B and C, a significant percentage of P/LP variants (28.5 and 31.8%, respectively) were identified in genes associated with increased risk of non-breast cancers for which established management guidelines exist (see Appendix 1 in Electronic Supplementary Material).

Perhaps surprisingly, 132 P/LP variants (11.7%) were unexpected clinically actionable findings (i.e., variants in genes for which patients had no suggestive personal or familial history and for which medical management guidelines exist). By panel, approximately 13 and 15% of the P/LP variants identified in groups B and C, respectively, consisted of these unexpected findings (Table 1).

**Table 1:** Unexpected findings with management guidelines by panel group

	Group A	Group B	Group C
Total variants	158	825 *n* (%)	148 *n* (%)
Clinically actionable findings unrelated to personal or family history	Non-breast cancer genes not assessed	110 (13.3)	22 (14.9)

**Fig. 4:**
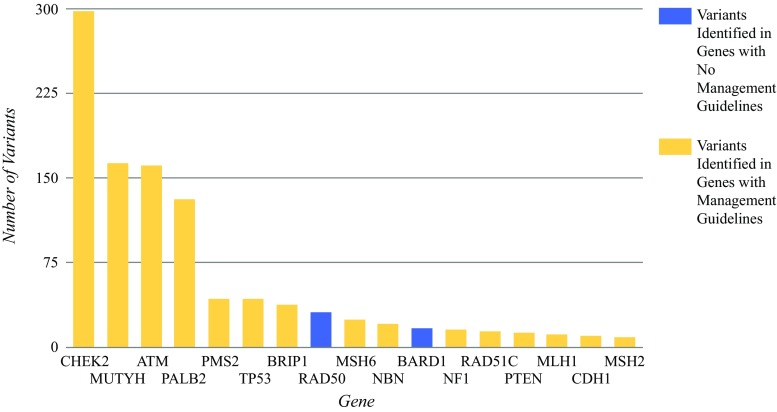
Most frequently identified pathogenic and likely pathogenic variants

Overall, the most common P/LP findings in *BRCA1/2*-negative patients were in *CHEK2* (27.6%), *MUTYH* (15%), *ATM* (14.9%), and *PALB2* (12.2%) (Fig. 4).

We also determined the number of patients with one or more variants of uncertain significance (VUS), excluding those with both VUS and P/LP variants. As expected, VUS rates were directly proportional to the number of genes analyzed. One VUS or more were found in 12.7% of the patients in group A, 31.6% of the patients in group B, and 49.6% of the patients in group C.

## Discussion

Clinicians face a multitude of options when ordering hereditary genetic testing for breast cancer risk, including single-gene tests such as those limited to *BRCA1/2*, multi-gene panels including genes associated with a high risk of breast cancer, panels for genes associated with multiple common cancer types (e.g., breast, gynecologic, and gastrointestinal), and comprehensive panels including genes associated with both common (e.g., prostate) and rare (e.g., sarcoma) cancers. Professional societies have published testing recommendations limited to specific genes such as *BRCA1/2* and those associated with well-described hereditary cancer conditions such as Li-Fraumeni (*TP53*) and Cowden syndrome (*PTEN*).^3^,^5^

The results of this report and others, however, suggest a trend toward ordering larger panels,^20^–^22^ which have the advantage of increased yield afforded by querying more genes. A recent study examining the yield of 348 multi-gene tests not limited to breast cancer found that the inclusion of moderate-risk genes increased only the positive rate of “historically high-risk” cancer genes.^26^ Another study, which assessed 90 HBOC patients, found that the positive rate roughly doubled when the panel included genes beyond *BRCA1/2*.^27^ Among clinic-based studies of *BRCA1/2*-negative patients, the prevalence of pathogenic variants in non-*BRCA* genes ranged from 3.7 to 16%.^14^–^17^,^28^–^31^

Our data support previous studies showing that expanded panel testing improves the identification of hereditary cancer risk for patients and their family members. We found that the use of expanded panels increased the number of clinically relevant results, many of which were not associated with personal or family history. In group A, the genes tested were limited to those associated with breast cancer, and therefore, all the variants identified were related to clinical or family history. In group B, however, patients were tested for genes associated with common cancer types (including breast, gynecologic, and gastrointestinal), and 13% of the P/LP variants were identified in genes that had published medical management recommendations but were not associated with the personal or family history. This number increased to 15% in group C, which represented the most comprehensive panels (Table 1). This proportion of unexpected findings is higher than previous estimates (8% in a cohort of 1046 *BRCA1/2*-negative individuals evaluated for HBOC).^32^

Our findings are consistent with prior studies suggesting that expanded panels increase diagnostic yield and furthermore showed that the vast majority of variants identified (92%) were associated with medical management guidelines that may alter clinical care. Additionally, our results suggest that breast cancer-specific panels may miss clinically actionable findings in genes associated with hereditary predispositions for other cancers.

Another important finding was that a considerable number of patients had more than one pathogenic variant. Of 44 patients with two or more clinically significant variants, 26 (59%) would not have been identified with a panel specific to breast cancer.

The findings showed P/LP variants in a variety of genes (Fig. 4), but most commonly in *CHEK2,* which accounted for 27.6% of our positive findings. This gene is associated with an increased risk for autosomal dominant adult-onset cancers including breast, colon, thyroid, and prostate cancers, and possibly others.^33^–^37^ These cancer risks, particularly the risks for breast cancer, depend on the variant and family history.^34^,^38^ Several studies have found that pathogenic variants in *CHEK2* are observed at a frequency higher than those of variants identified in other breast cancer-associated genes on multi-gene panels,^22^,^26^,^39^ which is consistent with our observations. One case example from our cohort was a woman who received a diagnosis of breast cancer at age 37. Her family history was significant for breast cancer in her mother (diagnosed when she was 47 years old) and maternal aunt (diagnosed when she was 49 years old). Although the patient had previous negative *BRCA1/2* testing, her clinician ordered a larger breast cancer panel based on her age at diagnosis and striking family history. The results showed a pathogenic variant in *CHEK2*.

Professional management guidelines include consideration of bilateral mastectomy or screening with breast magnetic resonance imaging (MRI) and colonoscopy, beginning at the age of 40 years.^3^,^5^ The use of *BRCA1/2* testing alone failed to provide an explanation for the breast cancers in this family. Expanded multi-gene testing showed clinically actionable results that not only explained the family history but also had implications for family members.

In this study, MUTYH heterozygotes accounted for 15% of our P/LP findings, likely due to the relatively high *MUTYH* carrier frequency among whites of Northern European descent. Approximately 1.5–2% of the individuals in this population carry one of two well-described founder variants: c.536A>G (p.Tyr179Cys) in exon 7 and c.1187G>A (p.Gly396Asp).^40^–^42^ It is known that *MUTYH* heterozygotes have an increased risk for colorectal cancer, and medical management and surveillance protocols have been developed.^4^ Colonoscopy every 5 years is recommended beginning at age 40 or 10 years earlier than the youngest age at diagnosis in an affected first-degree relative. An example from our cohort was a 40-year-old woman with a diagnosis of invasive ductal carcinoma and no family history of cancer. A 26-gene breast cancer panel identified a single pathogenic *MUTYH* variant. Although this result may not explain her early onset breast cancer diagnosis, it showed an increased risk for colon cancer, thereby allowing the implementation of screening guidelines.

The criticisms of multi-gene panels are well known. One criticism focuses on the increased risk of obtaining uncertain results lacking clear management guidelines, such as variants in genes with undefined cancer risks or P/LP variants in genes that lack established screening guidelines.^23^ Another concern is the risk of overestimating the clinical implications of a P/LP result in a low- to moderate-risk gene.^24^ A further criticism is the proportionate increase in the number of VUS for which management is unclear. Finally, multi-gene panels can require more extensive pre-test genetic counseling.^22^

Despite these limitations, the decreasing cost of genetic testing due to NGS technology has made multi-gene panels accessible to clinicians and patients. In this study, the price of ordering a genetic test was the same regardless of the number of genes on the panel, making test selection dependent only on patient and provider preferences. The majority (85.6%) of our cohort of 1085 patients were tested with broader panels that assessed risks for multiple cancer types (groups B and C). This result is consistent with the reported trend of providers ordering testing of more genes per patient.^25^

This study had a number of limitations. Although published management guidelines exist for the majority of positive variants identified, the utility of discovering these variants for post-test management was assumed but not assessed. Further analysis of patient outcomes is needed to clarify the actual clinical impact of panel testing. Because panel selection was at the discretion of the ordering provider, the genes analyzed varied by patient. This may have been influenced in part by the tendency of clinicians to order a large panel for patients with complicated clinical presentations. However, this was not assessed in our study. In addition, personal and family history information was limited to that on the test request form. Any relevant family history or secondary diagnosis was not analyzed. Finally, we were unable to assess ordering patterns by clinician type (genetic counselor, surgeons, oncologists, OB/gyn).

## Conclusion

Expanded panel testing increased the identification of P/LP findings related to non-breast cancers. Notably, most of these findings occurred in clinically actionable genes with published management recommendations.

The potential downsides of comprehensive panel testing must be weighed against the opportunity to discover actionable variants that may lead to earlier screening and detection, prevention, and decreased cancer morbidity. Pretest counseling should address the potential for incidental but actionable findings and findings that have no medical management guidelines. Finally, further analysis is needed to determine the clinical impact and patient outcomes associated with the identification of P/LP variants in non-*BRCA1/2* genes.

## Electronic supplementary material

Below is the link to the electronic supplementary material.

Supplementary material 1 (DOCX 59 kb)
