# Supplementary material for: Expanded Gene Panel Use for Women With Breast Cancer: Identification and Intervention Beyond Breast Cancer Risk
Source: Ann Surg Oncol. 2017 Aug 1;24(10):3060–6. doi: 10.1245/s10434-017-5963-7 (PMC5594040; doi:10.1245/s10434-017-5963-7)
Supplement: Supplementary file 1 — Supplementary material 1 (DOCX 59 kb) [file 10434_2017_5963_MOESM1_ESM.docx]

Supplemental Material

|  | **Gene** | **Cancer Risk (PMID)** | **Breast Management** | **Non-Breast Management** |
| --- | --- | --- | --- | --- |
|  | *ATM* | Breast^1^  Pancreatic^2^  Prostate^3^ | NCCN^®4^ | None |
|  | *BRCA1* | Breast^5^  Male breast^6^  Ovarian^7^  Prostate^8^  Pancreatic^9^ | - ASBS^10^ - NCCN^®4^ | NCCN^®4^ |
|  | *BRCA2* | Breast^11^  Male breast^6^  Ovarian^12^  Prostate^13^  Pancreatic^13^  Melanoma^13^ | - ASBS^10^ - NCCN^®4^ | NCCN^®4^ |
|  | *CDH1* | Breast^14^  Gastric^15^ | - ASBS^10^ - NCCN^®4^ | NCCN^®16^ |
|  | *CHEK2* | Breast^17^  Male breast^18^  Colorectal^19^  Prostate^20^  Thyroid^21^ | - ASBS^10^ - NCCN^®4^ | NCCN^®4^ |
|  | *NBN* | Breast^22^  Prostate^23^  Colorectal^24^  Hematologic malignancies^25^ | NCCN^®4^ | None |
|  | *NF1* | Breast^26^  CNS neoplasms^27^  Soft tissue sarcoma^28^  Others^29-31^ | NCCN^®4^ | Consensus Management Guidelines^32-34^ |
|  | *PALB2* | Breast^35,36^  Pancreatic^37^ | - ASBS^14^ - NCCN^®4^ | None |
|  | *PTEN* | Breast^38^  Thyroid^39^  Endometrial^40^  Colorectal^41^  Kidney^42^ | - ASBS^14^ - NCCN^®4^ | - NCCN^®4^ - Expert opinion^41^ |
|  | *STK11* | Breast^43,44^  Colorectal^44^  Ovarian^44^  Endometrial^44^  Gastric^44^  Pancreatic^43,44^  Lung^43,44^ | - ASBS^14^ - NCCN^®4^ | NCCN^®4^ |
|  | *TP53* | Breast^45^  Brain/CNS^46^  Sarcoma^45^  Adrenocortical carcinoma^47^  Ovarian^48^ Uterine^49^ Colon^50^ Gastric^51^ Pancreatic^51^  Lung^52^ Leukemia^53^ | - ASBS^14^ - NCCN^®4^ | - NCCN^®4^ - Expert opinion^54^ |
|  | *APC* | Colon^55, 56, 57^  Papillary thyroid^58^  Duodenum^59,60^  Gastric^61,62^  Brain/central nervous system^63^  Hepatoblastoma^64,65^ | - | NCCN^®66^ |
|  | *BAP1* | Uveal melanoma^67,68^  Cutaneous melanoma^67,68^  Mesothelioma^67,68^  Renal cell carcinoma^67,68^ | - | Expert opinion^67,69^ |
|  | *BMPR1A* | Colon^70,71^  Gastric^72^ | - | NCCN^®66^ |
|  | *BRIP1* | Ovarian^73-75^ | - | NCCN^®4^ |
|  | *CDC73* | Parathyroid carcinoma^76^ | - | Expert opinion^77,78^ |
|  | *CDK4* | Melanoma^79^ | - | Expert opinion^80-82^ |
|  | *CDKN2A* | Melanoma^82-84^  Pancreatic^82-84^ | - | Expert opinion^81,82,85^ |
|  | *DICER1* | Pleuropulmonary blastoma^86^  Thyroid^86^  Ovarian sex cord-stromal       tumors^86^  Pituitary blastoma^86^  Pineoblastoma^86^ | - | International       Pleuropulmonary Blastoma Registry Recommendations for Surveillance of High-Risk Children and Children with PPB^87^ |
|  | *EPCAM* | Colon^88^  Uterine^88^  Ovary^88^  Prostate^89^  Gastric^88^  Small intestine^88^  Hepatobiliary tract^88^  Urinary tract^88^  Pancreas^90^  Brain^88^ | - | NCCN^®66^ |
|  | *FH* | Renal  Leiomyosarcoma^91^ | - | Expert opinion^91^ |
|  | *FLCN* | Renal^92^ | - | Consensus management guideline^93^ |
|  | *KIT* | GIST^94,95^ | - | - NCCN^®96^ - Consensus management guidelines^97^ |
|  | *MAX* | Pheochromocytoma^98^ Paraganglioma^98^ | - | Consensus management guidelines^99^ |
|  | *MEN1* | Parathyroid^100^  Pitutary^100^  Pancreatic^100^ | - | - NCCN^®101^ - Consensus  management guidelines^100^ |
|  | *MET* | Papillary renal cell carcinoma^102^ | - | Expert opinion^102,103^ |
|  | *MLH1* | Colon^88^  Uterine^88^  Ovary^88^  Prostate^89^  Gastric^88^  Small intestine^88^  Hepatobiliary tract^88^  Urinary tract^88^  Pancreas^90^  Brain^88^ | - | NCCN^®66^ |
|  | *MSH2* | Colon^88^  Uterine^88^  Ovary^88^  Prostate^89^  Gastric^88^  Small intestine^88^  Hepatobiliary tract^88^  Urinary tract^88^  Pancreas^90^  Brain^88^ | - | NCCN^®66^ |
|  | *MSH6* | Colon^88^  Uterine^88^  Ovary^88^  Prostate^89^  Gastric^88^  Small intestine^88^  Hepatobiliary tract^88^  Urinary tract^88^  Pancreas^90^  Brain^88^ | - | NCCN^®66^ |
|  | *MUTYH* | Colon^104^  Duodenum^104^ | - | NCCN^®66^ |
|  | *NF2* | Schwannoma^105,106^  CNS tumors^105,106^ | - | Consensus management guidelines^107^ |
|  | *PDGFRA* | GIST^108,109,110^ | - | - NCCN^®96^ - Consensus management guidelines^97^ |
|  | *PMS2* | Colon^111^  Endometrium^111^  Ovary^111^  Prostate^111^  Stomach^111^  Small intestine^111^  Hepatobiliary tract^111^  Urinary tract^111^  Pancreas^111^  Brain^111^ | - | NCCN^®66^ |
|  | *PRKAR1A* | Psammomatous melanotic schwannoma^112^  Large-cell calcifying Sertoli cell tumors^112^ | - | Expert opinion^113^ |
|  | *PTCH1* | Basal cell carcinoma^114^ Medulloblastoma^114^ | - | Consensus management guidelines^115^ |
|  | *RB1* | Retinoblastoma^116^ Pinealoblastoma^117^  Osteosarcoma^116^  Soft tissue sarcomas^116^  Epithelial^116^  Melanoma^116^ | - | Consensus management guidelines^118^ |
|  | *RET* | Medullary thyroid carcinoma^119^  Pheochromocytoma^119^  Parathyroid hyperplasia^119^ | - | - NCCN^®101,120^ - Consensus        management       guidelines^121^ |
|  | *SDHA* | Paraganglioma^122^  Pheochromocytoma^122^  GIST^123^ | - | Consensus       management       guidelines^99^ |
|  | *SDHAF2* | Paraganglioma^122^  Pheochromocytoma^122^ | - | Consensus      management      guidelines^99^ |
|  | *SDHB* | Paraganglioma^122^  Pheochromocytoma^122^  GIST^122^  Kidney^124^ | - | Consensus       management       guidelines^99^ |
|  | *SDHC* | Paraganglioma^122^  Pheochromocytoma^122^  GIST^122^  Kidney^124^ | - | Consensus       management       guidelines^99^ |
|  | *SDHD* | Paraganglioma^122^  Pheochromocytoma^122^  GIST^124^ | - | Consensus       management       guidelines^99^ |
|  | *SMAD4* | Colon^70^  Gastric^125^ | - | - NCCN^®66^ - Consensus management guidelines^70,126^ |
|  | *SMARCB1* | Kidney^127^  Central nervous system^127^ | - | Expert opinion^128,129,130,131^ |
|  | *TMEM127* | Paraganglioma^132^  Pheochromocytoma^132^ | - | Consensus      management      guidelines^99^ |
|  | *TSC1* | Brain^133^  Kidney^133^ | - | Consensus management guidelines^134^ |
|  | *TSC2* | Brain^133^  Kidney^133^ | - | Consensus       management       guidelines^134^ |
|  | *VHL* | Kidney^135^  Pancreas^135^  Pheochromocytoma^135^  CNS^135^ | - | Consensus      management      guidelines^136^ |
|  | *WT1* | Kidney (Wilms tumor)^137,138^ | - | Expert opinion^139,140^ |

**Appendix 1.** Management Guidelines Resource

1. Renwick A, Thompson D, Seal S, Kelly P, Chagtai T, Ahmed M, et al. *ATM* mutations that cause ataxia-telangiectasia are breast cancer susceptibility alleles. *Nat Genet.* 2006;38(8):873-5.
2. Roberts NJ, Jiao Y, Yu J, Kopelovich L, Petersen GM, Bondy ML, et al. *ATM* mutations in patients with hereditary pancreatic cancer. *Cancer Discov.* 2012;2(1):41-6.
3. Pritchard CC, Mateo J, Walsh MF, De Sarkar N, Abida W, Beltran H, et al. Inherited DNA-repair gene mutations in men with metastatic prostate cancer. *N Engl J Med.* 2016;375(5):443-53.
4. NCCN Clinical Practice Guidelines in Oncology (NCCN Guidelines®) Genetic/Familial High-Risk Assessment: Breast and Ovarian. Version 2.2017. Accessed March 31, 2017.
5. Thompson D, Easton DF; Breast Cancer Linkage Consortium. Cancer incidence in *BRCA1* mutation carriers. *J Natl Cancer Inst.* 2002;94(18):1358-65.
6. Evans DG, Susnerwala I, Dawson J, Woodward E, Maher ER, Lalloo F. Risk of breast cancer in male *BRCA2* carriers. *J Med Genet.* 2010;47(10):710-1.
7. Antoniou A, Pharoah PD, Narod S, Risch HA, Eyfjord JE, Hopper JL, et al. Average risks of breast and ovarian cancer associated with *BRCA1* or *BRCA2* mutations detected in case Series unselected for family history: a combined analysis of 22 studies. *Am J Hum Genet.* 2003;72(5):1117-30.
8. Breast Cancer Linkage Consortium. Cancer risks in *BRCA2* mutation carriers. *J Natl Cancer Inst*. 1999;91(15):1310-6.
9. Iqbal J, Ragone A, Lubinski J, Lynch HT, Moller P, Ghadirian P, et al. The incidence of pancreatic cancer in *BRCA1* and *BRCA2* mutation carriers. *Br J Cancer.* 2012;107(12):2005-9.
10. The American Society of Breast Surgeons Consensus Guideline on Hereditary Genetic Testing for Patients With and Without Breast Cancer (revised March 14, 2017). Available: https://www.breastsurgeons.org/new_layout/about/statements/PDF_Statements/BRCA_Testing.pdf [accessed April 1, 2017].
11. King MC, Marks JH, Mandell JB; New York Breast Cancer Study Group. Breast and ovarian cancer risks due to inherited mutations in *BRCA1* and *BRCA2.* *Science.* 2003;302(5645):643-6.
12. Ford D, Easton DF, Stratton M, Narod S, Goldgar D, Devilee P, et al. Genetic heterogeneity and penetrance analysis of the *BRCA1* and *BRCA2* genes in breast cancer families. The Breast Cancer Linkage Consortium. *Am J Hum Genet.* 1998;62(3):676-89.
13. Breast Cancer Linkage Consortium. Cancer risks in *BRCA2* mutation carriers. *J Natl Cancer Inst.* 1999;91(15):1310-6.
14. Pharoah PD, Guilford P, Caldas C; International Gastric Cancer Linkage Consortium. Incidence of gastric cancer and breast cancer in *CDH1* (E-cadherin) mutation carriers from hereditary diffuse gastric cancer families. *Gastroenterology.* 2001;121(6):1348-53.
15. Richards FM, McKee SA, Rajpar MH, Cole TR, Evans DG, Jankowski JA, et al. Germline E-cadherin gene (*CDH1*) mutations predispose to familial gastric cancer and colorectal cancer. *Hum Mol Genet.* 1999;8(4):607-10.
16. NCCN Clinical Practice Guidelines in Oncology (NCCN Guidelines®) Gastric Cancer. Version 1.2017. Accessed March 31, 2017.
17. Cybulski C, Wokołorczyk D, Jakubowska A, Huzarski T, Byrski T, Gronwald J, et al. Risk of breast cancer in women with a *CHEK2* mutation with and without a family history of breast cancer. *J Clin Oncol.* 2011;29(28):3747-52.
18. Wasielewski M, den Bakker MA, van den Ouweland A, Meijer-van Gelder ME, Portengen H, Klijn JG, et al. *CHEK2* 1100delC and male breast cancer in the Netherlands. *Breast Cancer Res Treat.* 2009;116(2):397-400.
19. Xiang HP, Geng XP, Ge WW, Li H. Meta-analysis of *CHEK2* 1100delC variant and colorectal cancer susceptibility. *Eur J Cancer.* 2011;47(17):2546-51.
20. Han FF1, Guo CL, Liu LH. The effect of *CHEK2* variant I157T on cancer susceptibility: evidence from a meta-analysis. *DNA Cell Biol.* 2013;32(6):329-35.
21. Cybulski C, Górski B, Huzarski T, Masojć B, Mierzejewski M, Debniak T, et al. *CHEK2* is a multiorgan cancer susceptibility gene. *Am J Hum Genet.* 2004;75(6):1131-5.
22. Zhang B, Beeghly-Fadiel A, Long J, Zheng W. Genetic variants associated with breast-cancer risk: comprehensive research synopsis, meta-analysis, and epidemiological evidence. *Lancet Oncol.* 2011;12(5):477-88.
23. Cybulski C, Górski B, Debniak T, Gliniewicz B, Mierzejewski M, Masojć B, et al. *NBS1* is a prostate cancer susceptibility gene. *Cancer Res.* 2004;64(4):1215-9.
24. di Masi A, Antoccia A. *NBS1* heterozygosity and cancer risk. *Curr Genomics.* 2008;9(4):275-81.
25. Resnick IB, Kondratenko I, Pashanov E, Maschan AA, Karachunsky A, Togoev O, et al. 657del5 mutation in the gene for Nijmegen breakage syndrome (*NBS1*) in a cohort of Russian children with lymphoid tissue malignancies and controls. *Am J Med Genet A.* 2003;120A(2):174-9.
26. Madanikia SA, Bergner A, Ye X, Blakeley JO. Increased risk of breast cancer in women with NF1. *Am J Med Genet A.* 2012;158A(12):3056-60.
27. Evans DG, Baser ME, McGaughran J, Sharif S, Howard E, Moran A. Malignant peripheral nerve sheath tumours in neurofibromatosis 1. *J Med Genet.* 2002;39(5):311-4.
28. Zöller ME, Rembeck B, Odén A, Samuelsson M, Angervall L. Malignant and benign tumors in patients with neurofibromatosis type 1 in a defined Swedish population. *Cancer.* 1997;79(11):2125-31.
29. Gorgel A, Cetinkaya DD, Salgur F, Demirpence M, Yilmaz H, Karaman EH, et al. Coexistence of gastrointestinal stromal tumors (GISTs) and pheochromocytoma in three cases of neurofibromatosis type 1 (NF1) with a review of the literature. *Intern Med.* 2014;53(16):1783-9.
30. Seminog OO, Goldacre MJ. Risk of benign tumours of nervous system, and of malignant neoplasms, in people with neurofibromatosis: population-based record-linkage study. *Br J Cancer.* 2013;108(1):193-8.
31. Walker L, Thompson D, Easton D, Ponder B, Ponder M, Frayling I, Baralle D. A prospective study of neurofibromatosis type 1 cancer incidence in the UK. *Br J Cancer.* 2006 Jul 17;95(2):233-8.
32. Ferner RE, Gutmann DH. Neurofibromatosis type 1 (NF1): diagnosis and management. *Handb Clin Neurol.* 2013;115:939-55.
33. Ferner RE, Huson SM, Thomas N, Moss C, Willshaw H, Evans DG, et al. Guidelines for the diagnosis and management of individuals with neurofibromatosis 1. *J Med Genet.* 2007;44(2):81-8.
34. Hersh JH, American Academy of Pediatrics Committee on Genetics. Health supervision for children with neurofibromatosis. *Pediatrics.* 2008;121(3):633-42.
35. Antoniou AC, Casadei S, Heikkinen T, Barrowdale D, Pylkäs K, Roberts J, et al. Breast-cancer risk in families with mutations in *PALB2. N Engl J Med.* 2014;371(6):497-506.
36. Casadei S, Norquist BM, Walsh T, Stray S, Mandell JB, Lee MK, et al. Contribution of inherited mutations in the *BRCA2*-interacting protein *PALB2* to familial breast cancer. *Cancer Res*. 2011;71(6):2222-9.
37. Slater EP, Langer P, Niemczyk E, Strauch K, Butler J, Habbe N, et al. *PALB2* mutations in European familial pancreatic cancer families. *Clin Genet.* 2010;78(5):490-4.
38. Tan MH, Mester JL, Ngeow J, Rybicki LA, Orloff MS, Eng C. Lifetime cancer risks in individuals with germline *PTEN* mutations. *Clin Cancer Res.* 2012;18(2):400-7.
39. Hall JE, Abdollahian DJ, Sinard RJ. Thyroid disease associated with Cowden syndrome: A meta-analysis. *Head Neck.* 2013;35(8):1189-94.
40. Riegert-Johnson DL, Gleeson FC, Roberts M, Tholen K, Youngborg L, Bullock M, Boardman LA. Cancer and Lhermitte-Duclos disease are common in Cowden syndrome patients. *Hered Cancer Clin Pract.* 2010;8(1):6.
41. Stanich PP, Pilarski R, Rock J, Frankel WL, El-Dika S, Meyer MM. Colonic manifestations of *PTEN* hamartoma tumor syndrome: case series and systematic review. *World J Gastroenterol.* 2014;20(7):1833-8.
42. Mester J, Charis E. *PTEN* hamartoma tumor syndrome. *Handb Clin Neurol.* 2015;132:129-37.
43. van Lier MG, Wagner A, Mathus-Vliegen EM, Kuipers EJ, Steyerberg EW, van Leerdam ME. High cancer risk in Peutz-Jeghers syndrome: a systematic review and surveillance recommendations. *Am J Gastroenterol.* 2010;105(6):1258-64.
44. Hearle N, Schumacher V, Menko FH, Olschwang S, Boardman LA, Gille JJ, et al. Frequency and spectrum of cancers in the Peutz-Jeghers syndrome. *Clin Cancer Res.* 2006;12(10):3209-15.
45. Chompret A, Brugières L, Ronsin M, Gardes M, Dessarps-Freichey F, Abel A, et al. *P53* germline mutations in childhood cancers and cancer risk for carrier individuals. *Br J Cancer.* 2000;82(12):1932-7.
46. Ruijs MW, Verhoef S, Rookus MA, Pruntel R, van der Hout AH, Hogervorst FB, et al. *TP53* germline mutation testing in 180 families suspected of Li-Fraumeni syndrome: mutation detection rate and relative frequency of cancers in different familial phenotypes. *J Med Genet.* 2010;47(6):421-8.
47. Bougeard G, Renaux-Petel M, Flaman JM, Charbonnier C, Fermey P, Belotti M, et al. Revisiting Li-Fraumeni syndrome from *TP53* mutation carriers. *J Clin Oncol*. 2015;33(21):2345-52.
48. Olivier M, Goldgar DE, Sodha N, Ohgaki H, Kleihues P, Hainaut P, Eeles RA. Li-Fraumeni and related syndromes: correlation between tumor type, family structure, and *TP53* genotype. *Cancer Res.* 2003;63(20):6643-50.
49. Schneider K, Zelley K, Nichols KE, Garber J. Li-Fraumeni Syndrome. In: Pagon RA, Adam MP, Ardinger HH, Wallace SE, Amemiya A, Bean LJH, et al, editors. GeneReviews® [Internet]. Seattle (WA): University of Washington, Seattle; 1993–2017. Updated April 11, 2013. Available at: https://www.ncbi.nlm.nih.gov/books/NBK1311/
50. Wong P, Verselis SJ, Garber JE, Schneider K, DiGianni L, Stockwell DH, et al. Prevalence of early onset colorectal cancer in 397 patients with classic Li-Fraumeni syndrome. *Gastroenterology.* 2006;130(1):73-9.
51. Masciari S, Dewanwala A, Stoffel EM, Lauwers GY, Zheng H, Achatz MI, et al. Gastric cancer in individuals with Li-Fraumeni syndrome. *Genet Med.* 2011;13(7):651-7.
52. Tinat J, Bougeard G, Baert-Desurmont S, Vasseur S, Martin C, Bouvignies E, et al. 2009 version of the Chompret criteria for Li Fraumeni syndrome. *J Clin Oncol.* 2009;27(26):e108-9.
53. Giacomazzi CR, Giacomazzi J, Netto CB, Santos-Silva P, Selistre SG, Maia AL, et al. Pediatric cancer and Li-Fraumeni/Li-Fraumeni-like syndromes: a review for the pediatrician. *Rev Assoc Med Bras (1992).* 2015;61(3):282-9.
54. Villani A, Tabori U, Schiffman J, Shlien A, Beyene J, Druker H, et al. Biochemical and imaging surveillance in germline *TP53* mutation carriers with Li-Fraumeni syndrome: a prospective observational study. *Lancet Oncol.* 2011;12(6):559-67.
55. Half E, Bercovich D, Rozen P. Familial adenomatous polyposis. *Orphanet J Rare Dis.* 2009;4:22.
56. Neklason DW, Stevens J, Boucher KM, Kerber RA, Matsunami N, Barlow J, et al. American founder mutation for attenuated familial adenomatous polyposis. *Clin Gastroenterol Hepatol.* 2008;6(1):46-52.
57. Petersen GM, Slack J, Nakamura Y. Screening guidelines and premorbid diagnosis of familial adenomatous polyposis using linkage. *Gastroenterology.* 1991;100(6):1658-64.
58. Septer S, Slowik V, Morgan R, Dai H, Attard T. Thyroid cancer complicating familial adenomatous polyposis: mutation spectrum of at-risk individuals. *Hered Cancer Clin Pract.* 2013;11(1):13.
59. Groves CJ, Saunders BP, Spigelman AD, Phillips RK. Duodenal cancer in patients with familial adenomatous polyposis (FAP): results of a 10 year prospective study. *Gut.* 2002;50(5):636-41.
60. Biasco G, Pantaleo MA, Di Febo G, Calabrese C, Brandi G, Bülow S. Risk of duodenal cancer in patients with familial adenomatous polyposis. *Gut*. 2004;53(10):1547.
61. Bianchi LK, Burke CA, Bennett AE, Lopez R, Hasson H, Church JM. Fundic gland polyp dysplasia is common in familial adenomatous polyposis. *Clin Gastroenterol Hepatol.* 2008;6(2):180-5.
62. Spigelman AD, Williams CB, Talbot IC, Domizio P, Phillips RK. Upper gastrointestinal cancer in patients with familial adenomatous polyposis. *Lancet.* 1989;2(8666):783-5.
63. Attard TM, Giglio P, Koppula S, Snyder C, Lynch HT. Brain tumors in individuals with familial adenomatous polyposis: a cancer registry experience and pooled case report analysis. *Cancer.* 2007;109(4):761-6.
64. Giardiello FM, Petersen GM, Brensinger JD, Luce MC, Cayouette MC, Bacon J, et al. Hepatoblastoma and APC gene mutation in familial adenomatous polyposis. *Gut.* 1996;39(6):867-9.
65. Hughes LJ, Michels VV. Risk of hepatoblastoma in familial adenomatous polyposis. *Am J Med Genet.* 1992;43(6):1023-5.
66. NCCN Clinical Practice Guidelines in Oncology (NCCN Guidelines®) Genetic/Familial High-Risk Assessment: Colorectal. Version 2.2016. Accessed March 31, 2017.
67. Rai K, Pilarski R, Cebulla CM, Abdel-Rahman MH. Comprehensive review of *BAP1* tumor predisposition syndrome with report of two new cases. *Clin Genet.* 2016;89(3):285-94.
68. Popova T, Hebert L, Jacquemin V, Gad S, Caux-Moncoutier V, Dubois-d'Enghien C, et al. Germline BAP1 mutations predispose to renal cell carcinomas. *Am J Hum Genet.* 2013;92(6):974-80.
69. Battaglia A. The Importance of Multidisciplinary Approach in Early Detection of *BAP1* Tumor Predisposition Syndrome: Clinical Management and Risk Assessment. *Clin Med Insights Oncol.* 2014;8:37-47.
70. Syngal S, Brand RE, Church JM, Giardiello FM, Hampel HL, Burt RW; American College of Gastroenterology. ACG clinical guideline: Genetic testing and management of hereditary gastrointestinal cancer syndromes. *Am J Gastroenterol.* 2015;110(2):223-62.
71. Chow E, Macrae F. A review of juvenile polyposis syndrome. *J Gastroenterol Hepatol.* 2005;20(11):1634-40.
72. Pollock J, Welsh JS. Clinical cancer genetics: Part I: Gastrointestinal. *Am J Clin Oncol.* 2011;34(3):332-6.
73. Norquist BM, Harrell MI, Brady MF, Walsh T, Lee MK, Gulsuner S, et al. Inherited Mutations in Women With Ovarian Carcinoma. *JAMA Oncol.* 2016;2(4):482-90.
74. Rafnar T, Gudbjartsson DF, Sulem P, Jonasdottir A, Sigurdsson A, Jonasdottir A, et al. Mutations in *BRIP1* confer high risk of ovarian cancer. *Nat Genet.* 2011;43(11):1104-7.
75. Seal S, Thompson D, Renwick A, Elliott A, Kelly P, Barfoot R, et al. Truncating mutations in the Fanconi anemia J gene *BRIP1* are low-penetrance breast cancer susceptibility alleles. *Nat Genet.* 2006;38(11):1239-41.
76. Sharretts JM, Kebebew E, Simonds WF. Parathyroid cancer. *Semin Oncol.* 2010;37(6):580-90.
77. Li Y, Simonds WF. Endocrine neoplasms in familial syndromes of hyperparathyroidism. *Endocr Relat Cancer.* 2015;23(6):R229-47.
78. Jackson MA, Rich TA, Hu MI, Perrier ND, Waguespack SG. *CDC73*-Related Disorders. In: Pagon RA, Adam MP, Ardinger HH, Wallace SE, Amemiya A, Bean LJH, et al, editors. GeneReviews® [Internet]. Seattle (WA): University of Washington, Seattle; 1993-2017. Updated January 15, 2015. Available at: https://www.ncbi.nlm.nih.gov/books/NBK3789/
79. Goldstein AM, Struewing JP, Chidambaram A, Fraser MC, Tucker MA. Genotype-phenotype relationships in U.S. melanoma-prone families with *CDKN2A* and *CDK4* mutations. *J Natl Cancer Inst.* 2000;92(12):1006-10.
80. Soura E, Eliades PJ, Shannon K, Stratigos AJ, Tsao H. Hereditary melanoma: Update on syndromes and management: Genetics of familial atypical multiple mole melanoma syndrome. *J Am Acad Dermatol.* 2016;74(3):395-407.
81. Potrony M, Badenas C, Aguilera P, Puig-Butille JA, Carrera C, Malvehy J, Puig S. Update in genetic susceptibility in melanoma. *Ann Transl Med.* 2015;3(15):210.
82. Aoude LG, Wadt KA, Pritchard AL, Hayward NK. Genetics of familial melanoma: 20 years after *CDKN2A*. *Pigment Cell Melanoma Res.* 2015;28(2):148-60.
83. Begg CB, Orlow I, Hummer AJ, Armstrong BK, Kricker A, Marrett LD, et al. Lifetime risk of melanoma in *CDKN2A* mutation carriers in a population-based sample. *J Natl Cancer Inst.* 2005;97(20):1507-15.
84. Vasen HF, Gruis NA, Frants RR, van Der Velden PA, Hille ET, Bergman W. Risk of developing pancreatic cancer in families with familial atypical multiple mole melanoma associated with a specific 19 deletion of *p16* (*p16-Leiden*). *Int J Cancer.* 2000;87(6):809-11.
85. Soura E, Eliades PJ, Shannon K, Stratigos AJ, Tsao H. Hereditary melanoma: Update on syndromes and management: Genetics of familial atypical multiple mole melanoma syndrome. *J Am Acad Dermatol.* 2016;74(3):395-407.
86. Slade I, Bacchelli C, Davies H, Murray A, Abbaszadeh F, Hanks S, et al. *DICER1* syndrome: clarifying the diagnosis, clinical features and management implications of a pleiotropic tumour predisposition syndrome. *J Med Genet.* 2011;48(4):273-8.
87. The International Pleuropulmonary Blastoma Registry Surveillance Recommendations for High-Risk Children and Children with PPB. Available: http://www.ppbregistry.org/health-professionals/recommendations/surveillance [accessed April 13, 2017].
88. Kastrinos F, Stoffel EM. History, genetics, and strategies for cancer prevention in Lynch syndrome. *Clin Gastroenterol Hepatol.* 2014;12(5):715-27.
89. Maillard F, Manouvrier S, Biardeau X, Ouzzane A, Villers A. [Lynch syndrome and risk of prostate cancer; review of the literature]. *Prog Urol.* 2015;25(5):225-32.
90. Kastrinos F, Mukherjee B, Tayob N, Wang F, Sparr J, Raymond VM, et al. Risk of pancreatic cancer in families with Lynch syndrome. *JAMA.* 2009;302(16):1790-5.
91. Menko FH, Maher ER, Schmidt LS, Middelton LA, Aittomäki K, Tomlinson I, et al. Hereditary leiomyomatosis and renal cell cancer (HLRCC): renal cancer risk, surveillance and treatment. *Fam Cancer.* 2014;13(4):637-44.
92. Houweling AC, Gijezen LM, Jonker MA, van Doorn MB, Oldenburg RA, van Spaendonck-Zwarts KY, et al. Renal cancer and pneumothorax risk in Birt-Hogg-Dubé syndrome; an analysis of 115 *FLCN* mutation carriers from 35 BHD families. *Br J Cancer.* 2011;105(12):1912-9.
93. Menko FH, van Steensel MA, Giraud S, Friis-Hansen L, Richard S, Ungari S, et al. Birt-Hogg-Dubé syndrome: diagnosis and management. *Lancet Oncol.* 2009;10(12):1199-206.
94. Kleinbaum EP, Lazar AJ, Tamborini E, Mcauliffe JC, Sylvestre PB, Sunnenberg TD, et al. Clinical, histopathologic, molecular and therapeutic findings in a large kindred with gastrointestinal stromal tumor. *Int J Cancer.* 2008;122(3):711-8.
95. Antonescu CR. Gastrointestinal stromal tumor (GIST) pathogenesis, familial GIST, and animal models. *Semin Diagn Pathol.* 2006;23(2):63-9.
96. von Mehren M, Randall RL, Benjamin RS, Boles S, Bui MM, Conrad EU, et al. NCCN Clinical Practice Guidelines in Oncology (NCCN Guidelines®) Soft Tissue Sarcoma. Version 2.2017. Accessed April 13, 2017.
97. ESMO/European Sarcoma Network Working Group. Gastrointestinal stromal tumours: ESMO Clinical Practice Guidelines for diagnosis, treatment and follow-up. *Ann Oncol.* 2014;25 Suppl 3:iii21-6.
98. Burnichon N, Cascón A, Schiavi F, Morales NP, Comino-Méndez I, Abermil N, et al. *MAX* mutations cause hereditary and sporadic pheochromocytoma and paraganglioma. *Clin Cancer Res.* 2012;18(10):2828-37.
99. Lenders JW, Duh QY, Eisenhofer G, Gimenez-Roqueplo AP, Grebe SK, Murad MH, et al. Pheochromocytoma and paraganglioma: an endocrine society clinical practice guideline. *J Clin Endocrinol Metab.* 2014;99(6):1915-42.
100. Thakker RV1, Newey PJ, Walls GV, Bilezikian J, Dralle H, Ebeling PR, et al. Clinical practice guidelines for multiple endocrine neoplasia type 1 (MEN1). *J Clin Endocrinol Metab.* 2012;97(9):2990-3011.
101. Kulke MH, Shah MH, Benson AB, Bergsland E, Berlin JD, Besh SA, et al. NCCN Clinical Practice Guidelines in Oncology (NCCN Guidelines®) Neuroendocrine Tumors. Version 2.2017. Accessed April 13, 2017.
102. Adeniran AJ, Shuch B, Humphrey PA. Hereditary Renal Cell Carcinoma Syndromes: Clinical, Pathologic, and Genetic Features. *Am J Surg Pathol.* 2015;39(12):e1-e18.
103. Mustafa S, Jadidi N, Faraj SF, Rodriguez R. Case of hereditary papillary renal cell carcinoma. *J Community Hosp Intern Med Perspect.* 2012;1(4).
104. Jasperson KW, Tuohy TM, Neklason DW, Burt RW. Hereditary and familial colon cancer. *Gastroenterology.* 2010;138(6):2044-58.
105. Lloyd SK, Evans DG. Neurofibromatosis type 2 (NF2): diagnosis and management. *Handb Clin Neurol.* 2013;115:957-67.
106. Baser ME, Friedman JM, Joe H, Shenton A, Wallace AJ, Ramsden RT, Evans DG. Empirical development of improved diagnostic criteria for neurofibromatosis 2. *Genet Med.* 2011;13(6):576-81.
107. Blakeley JO, Evans DG, Adler J, Brackmann D, Chen R, Ferner RE, et al. Consensus recommendations for current treatments and accelerating clinical trials for patients with neurofibromatosis type 2. *Am J Med Genet A*. 2012;158A(1):24-41.
108. Ricci R, Martini M, Cenci T, Carbone A, Lanza P, Biondi A,  et al. *PDGFRA*-mutant syndrome. *Mod Pathol.* 2015;28(7):954-64.
109. de Raedt T, Cools J, Debiec-Rychter M, Brems H, Mentens N, Sciot R, et al. Intestinal neurofibromatosis is a subtype of familial GIST and results from a dominant activating mutation in *PDGFRA*. *Gastroenterology.* 2006;131(6):1907-12.
110. Chompret A, Kannengiesser C, Barrois M, Terrier P, Dahan P, Tursz T, et al. *PDGFRA* germline mutation in a family with multiple cases of gastrointestinal stromal tumor. *Gastroenterology.* 2004;126(1):318-21.
111. Goodenberger ML, Thomas BC, Riegert-Johnson D, Boland CR, Plon SE, Clendenning M, et al. *PMS2* monoallelic mutation carriers: the known unknown. *Genet Med.* 2016;18(1):13-9.
112. Stratakis CA, Kirschner LS, Carney JA. Clinical and molecular features of the Carney complex: diagnostic criteria and recommendations for patient evaluation. *J Clin Endocrinol Metab.* 2001;86(9):4041-6.
113. Siordia JA. Medical and Surgical Management of Carney Complex. *J Card Surg.* 2015;30(7):560-7.
114. Fujii K, Miyashita T. Gorlin syndrome (nevoid basal cell carcinoma syndrome): update and literature review. *Pediatr Int.* 2014;56(5):667-74.
115. Bree AF, Shah MR; BCNS Colloquium Group. Consensus statement from the first international colloquium on basal cell nevus syndrome (BCNS). *Am J Med Genet A*. 2011;155A(9):2091-7.
116. Dommering CJ, Marees T, van der Hout AH, Imhof SM, Meijers-Heijboer H, Ringens PJ, et al. *RB1* mutations and second primary malignancies after hereditary retinoblastoma. *Fam Cancer.* 2012;11(2):225-33.
117. Sato K, Kubota T. Pathology of pineal parenchymal tumors. *Prog Neurol Surg.* 2009;23:12-25.
118. Canadian Retinoblastoma Society. National Retinoblastoma Strategy Canadian Guidelines for Care: Stratégie thérapeutique du rétinoblastome guide clinique canadien. *Can J Ophthalmol.* 2009;44 Suppl 2:S1-88.
119. Moline J, Eng C. Multiple endocrine neoplasia type 2: an overview. *Genet Med.* 2011;13(9):755-64.
120. Haddad RI, Lydiatt WM, Bischoff L, Busaidy NL, Byrd D, Callender G, et al. NCCN Clinical Practice Guidelines in Oncology (NCCN Guidelines®) Thyroid Carcinoma.  Version 1.2017. Accessed April 13, 2017.
121. Wells SA Jr, Asa SL, Dralle H, Elisei R, Evans DB, Gagel RF, et al. Revised American Thyroid Association guidelines for the management of medullary thyroid carcinoma. *Thyroid.* 2015;25(6):567-610.
122. Bardella C, Pollard PJ, Tomlinson I. *SDH* mutations in cancer. *Biochim Biophys Acta.* 2011;1807(11):1432-43.
123. Italiano A, Chen CL, Sung YS, Singer S, DeMatteo RP, LaQuaglia MP, et al. *SDHA* loss of function mutations in a subset of young adult wild-type gastrointestinal stromal tumors. *BMC Cancer.* 2012;12:408.
124. Ricketts CJ, Shuch B, Vocke CD, Metwalli AR, Bratslavsky G, Middelton L, et al. Succinate dehydrogenase kidney cancer: an aggressive example of the Warburg effect in cancer. *J Urol.* 2012;188(6):2063-71.
125. Pollock J, Welsh JS. Clinical cancer genetics: Part I: Gastrointestinal. *Am J Clin Oncol.* 2011;34(3):332-6.
126. Faughnan ME, Palda VA, Garcia-Tsao G, Geisthoff UW, McDonald J, Proctor DD, et al. International guidelines for the diagnosis and management of hereditary haemorrhagic telangiectasia. *J Med Genet.* 2011;48(2):73-87.
127. Heck JE1, Lombardi CA, Cockburn M, Meyers TJ, Wilhelm M, Ritz B. Epidemiology of rhabdoid tumors of early childhood. *Pediatr Blood Cancer*. 2013;60(1):77-81.
128. Blakeley JO, Plotkin SR. Therapeutic advances for the tumors associated with neurofibromatosis type 1, type 2, and schwannomatosis. *Neuro Oncol.* 2016;18(5):624-38.
129. Koontz NA, Wiens AL, Agarwal A, Hingtgen CM, Emerson RE, Mosier KM. Schwannomatosis: the overlooked neurofibromatosis? *AJR Am J Roentgenol.* 2013;200(6):W646-53.
130. Reddy RG, Banda VR, Gunadal S, Banda NR. A rare occurrence and management of familial schwannomatosis. *BMJ Case Rep*. 2013;2013. pii: bcr2013008843
131. Teplick A, Kowalski M, Biegel JA, Nichols KE. Educational paper: screening in cancer predisposition syndromes: guidelines for the general pediatrician. *Eur J Pediatr.* 2011;170(3):285-94.
132. Abermil N, Guillaud-Bataille M, Burnichon N, Venisse A, Manivet P, Guignat L, et al. *TMEM127* screening in a large cohort of patients with pheochromocytoma and/or paraganglioma. *J Clin Endocrinol Metab.* 2012;97(5):E805-9.
133. Islam MP1 Roach ES. Tuberous sclerosis complex. *Handb Clin Neurol.* 2015;132:97-109.
134. Hinton RB, Prakash A, Romp RL, Krueger DA, Knilans TK; International Tuberous Sclerosis Consensus Group. Cardiovascular manifestations of tuberous sclerosis complex and summary of the revised diagnostic criteria and surveillance and management recommendations from the International Tuberous Sclerosis Consensus Group. *J Am Heart Assoc.* 2014;3(6):e001493.
135. Maher ER, Neumann HP, Richard S. von Hippel-Lindau disease: a clinical and scientific review. *Eur J Hum Genet.* 2011;19(6):617-23.
136. Binderup ML, Bisgaard ML, Harbud V, Møller HU, Gimsing S, Friis-Hansen L, et al. Von Hippel-Lindau disease (vHL). National clinical guideline for diagnosis and surveillance in Denmark. 3rd edition. *Dan Med J.* 2013;60(12):B4763.
137. Kaneko Y, Okita H, Haruta M, Arai Y, Oue T, Tanaka Y, Horie H, et al. A high incidence of *WT1* abnormality in bilateral Wilms tumours in Japan, and the penetrance rates in children with *WT1* germline mutation. *Br J Cancer.* 2015;112(6):1121-33.
138. Pritchard-Jones K. Controversies and advances in the management of Wilms' tumour. *Arch Dis Child.* 2002;87(3):241-4.
139. Szychot E, Apps J, Pritchard-Jones K. Wilms' tumor: biology, diagnosis and treatment. *Transl Pediatr.* 2014;3(1):12-24.
140. Wu HY, Snyder HM 3rd, D'Angio GJ. Wilms' tumor management. *Curr Opin Urol.* 2005;15(4):273-6.
